# Marking and Quantifying IL-17A-Producing Cells *In Vivo*

**DOI:** 10.1371/journal.pone.0039750

**Published:** 2012-06-29

**Authors:** April E. Price, R. Lee Reinhardt, Hong-Erh Liang, Richard M. Locksley

**Affiliations:** 1 Howard Hughes Medical Institute, University of California San Francisco, San Francisco, California, United States of America; 2 Department of Medicine, University of California San Francisco, San Francisco, California, United States of America; 3 Department of Microbiology and Immunology, University of California San Francisco, San Francisco, California, United States of America; McGill University Health Center, Canada

## Abstract

Interleukin (IL)-17A plays an important role in host defense against a variety of pathogens and may also contribute to the pathogenesis of autoimmune diseases. However, precise identification and quantification of the cells that produce this cytokine *in vivo* have not been performed. We generated novel IL-17A reporter mice to investigate expression of IL-17A during *Klebsiella pneumoniae* infection and during experimental autoimmune encephalomyelitis, conditions previously demonstrated to potently induce IL-17A production. In both settings, the majority of IL-17A was produced by non-CD4^+^ T cells, particularly γδ T cells, but also invariant NKT cells and other CD4^−^CD3ε^+^ cells. As measured in dual-reporter mice, IFN-γ-producing Th1 cells greatly outnumbered IL-17A-producing Th17 cells throughout both challenges. Production of IL-17A by cells from unchallenged mice or by non-T cells under any condition was not evident. Administration of IL-1β and/or IL-23 elicited rapid production of IL-17A by γδ T cells, invariant NKT cells and other CD4^−^CD3ε^+^ cells *in vivo*, demonstrating that these cells are poised for rapid cytokine production and likely comprise the major sources of this cytokine during acute immunologic challenges.

## Introduction

The cytokine interleukin (IL)-17A has a key role in immunity, inducing the release of a variety of inflammatory cytokines as well as chemokines that can mediate the rapid recruitment of neutrophils. Studies with knockout mice and neutralizing antibodies have revealed a role for IL-17A in immunity to various bacterial and fungal infections and also in the induction and propagation of several autoimmune diseases [1]. In humans, genetic deficiencies in IL-17 receptor A or IL-17F are associated with susceptibility to mucocutaneous infection with *Candida albicans* and, to a lesser extent, *Staphylococcus aureus* [2]. Autoantibodies to IL-17A and IL-17F are seen in patients with mutations in the autoimmune regulator (*AIRE*) and may contribute to mucocutaneous candidiasis [3]. Patients with hyper-IgE syndrome associated with mutations in Stat3 have deficits in the induction of IL-17-producing CD4^+^ T (Th17) cells that correlate with recurrent bacterial and fungal infections [4]. Polymorphisms in the gene encoding the receptor to IL-23, a cytokine implicated in the generation and maintenance of Th17 cells and in the promotion of IL-17 secretion from innate cells [5], [6], [7], [8], are associated with protection against the development of inflammatory bowel disease [9]. A more complete understanding of the biology of IL-17A, including the temporal and cell-specific patterns of expression, could aid in the generation of effective therapeutics targeted towards these infectious and autoimmune conditions.

Although early work focused on IL-17A production by Th17 cells, more recent studies suggest important contributions by innate-like lymphocytes, including γδ T cells and NKT cells [10]. However, most reports use *ex vivo* restimulation to identify IL-17A-producing cells, and thus potentially alter the pattern of cytokine secretion that occurs *in vivo*. Several groups have generated IL-17F reporter mice in order to bypass the need for restimulation, but while IL-17A and IL-17F are often co-expressed, they are differentially induced in certain models and can have distinct functional roles [11], [12], [13]. More recently, IL-17A-cre reporter mice were developed to permanently mark cells that have activated the IL-17A locus, thus facilitating fate-tracking, and an IL-17A-eGFP knockin mouse was used to track Th17 cells during tolerance induced by a CD3-specific antibody and in mouse models of sepsis [14], [15]. Studies using IL-17F reporter mice and IL-17A-cre mice, as well as studies using adoptive transfer [16], [17], have raised the possibility that Th17 cells have an unstable phenotype, such that they lose the capacity to produce IL-17 and begin to produce interferon (IFN)-γ.

Here, we generated IL-17A reporter mice and used these mice to examine the expression of IL-17A at rest, after bacterial challenge, and during the development of autoimmune encephalitis. To establish the relationships between IL-17A-producing cells and IFN-γ-producing cells, we additionally crossed these IL-17A reporter mice to mice with an IFN-γ reporter allele. Using these dual-reporter mice, we quantified the numbers and types of cells that produce these cytokines *in vivo* without the need for *ex vivo* restimulation.

## Results

### Generation and validation of Smart-17A reporter mice

**Figure 1:**
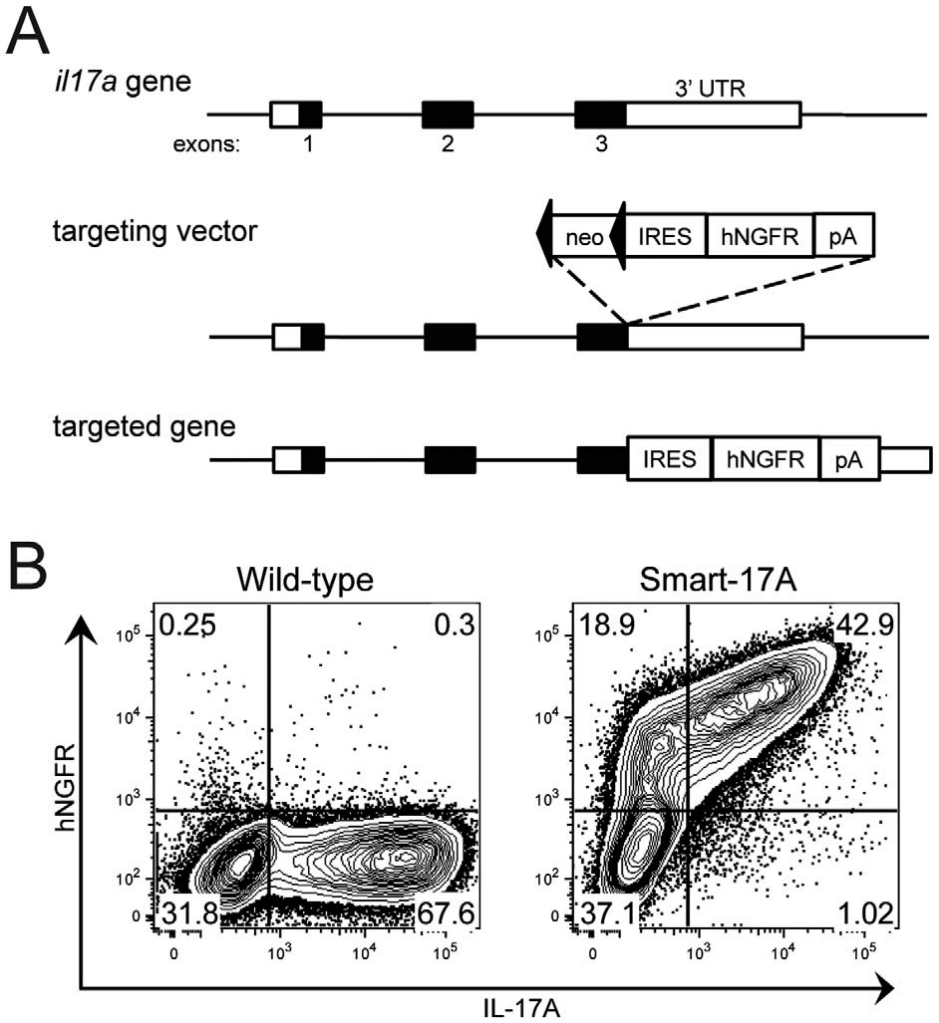
Generation of Smart-17A mice. (A) Targeting strategy for the *il17a* locus. For detailed description, see Materials and Methods. (B) CD4^+^ T cells were isolated from wild-type or Smart-17A mice and polarized under Th17 conditions for 4 days. hNGFR was detected using a surface antibody and IL-17A was assayed using intracellular cytokine staining after restimulation. A representative flow cytometry plot is shown from >5 comparable experiments.

To assess IL-17A expression *in vitro* and *in vivo*, we generated IL-17A reporter mice, termed Smart (Surface marker for transcription)-17A mice (Figure 1A). In these mice, the 3′ untranslated region (UTR) of the *il17a* gene was modified to include an internal ribosomal entry site (IRES) followed by a non-signaling form of the human nerve growth factor (hNGFR) gene, resulting in IRES-mediated translation of hNGFR when the IL-17A locus is activated. We verified the efficacy of the Smart-17A allele by demonstrating that hNGFR expression was specifically induced in CD4^+^ T cells *in vitro* only under Th17 polarizing conditions and that intracellular IL-17A was found almost entirely within the hNGFR^+^ population (Figure 1B, Figure S1A). Thus, the hNGFR reporter accurately marks 98% of Th17 cells identified using standard methods of *in vitro* restimulation and intracellular cytokine staining. Cells with the brightest staining for intracellular IL-17A were also those with the highest mean fluorescence intensity (MFI) for the hNGFR reporter. Approximately 30% of cells were hNGFR^+^ but negative for intracellular IL-17A (Figure 1B). These cells tended to have the lowest MFI for hNGFR, consistent with their identification as cells that had previously secreted IL-17A and continued to be marked by the surface reporter. The half-life of the reporter on the cell surface was approximately 24–48 hours as assessed by decay under *in vitro* conditions (Figure S1B). Taken together, these results demonstrate that the Smart-17A reporter mouse sensitively and accurately marks cells that are induced to express IL-17A.

### IL-17A expression in naïve mice

**Figure 2:**
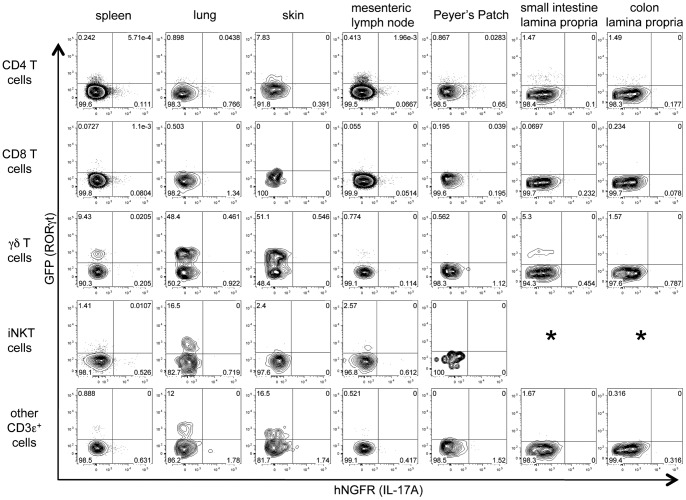
IL-17A expression in resting mice. Cells were isolated from indicated organs of Smart-17A/RORγt reporter mice and levels of hNGFR were assayed. Populations were gated as described in Figure S2. All gates were set using a wild-type mouse as a negative control. * denotes cell populations that were too few in number to reliably assess marker expression. Representative flow cytometry plots are shown from 1 of 3 comparable experiments, each including 2–3 mice.

To characterize IL-17A expression *in vivo*, we crossed Smart-17A mice to RORγt-GFP reporter mice [18]. Expression of the transcription factor RORγt has been shown to accurately identify both Th17 cells [19] and other lymphoid IL-17-producing cells [10]. We anticipated that the inclusion of this second reporter would enhance the detection of rare populations of IL-17A-expressing cells by their concordant expression of RORγt. We first looked at CD3ε^+^ cell populations in multiple organs of naïve mice for evidence of RORγt and IL-17A expression (Figure 2). CD3ε^+^ cells were gated as indicated in Figure S2. Invariant (i)NKT cells were identified using a tetramer loaded with PBS-57 (an analogue of α-galactosylceramide provided by the NIH tetramer facility), and “other CD3ε+ cells” were defined as cells that were CD3ε+ but negative for CD4, CD8, the γδ T cell receptor (TCR) and the CD1d-tetramer.

As previously reported, we observed constitutive expression of the RORγt GFP reporter in CD4^+^ T cells in the lamina propria of the small intestine and colon [19]. We also identified populations of GFP^+^ γδ T cells in the spleen, lung, skin and small intestine, GFP^+^ iNKT cells in the lung, and GFP^+^ other CD3ε^+^ cells in the lung and skin. Despite recovery of these RORγt^+^ cells, however, we did not observe hNGFR^+^ cells among the CD3ε^+^ cell populations in any of the examined organs. In all populations, the small percentage of cells within the hNGFR^+^ gate was similar to background levels present in naïve wild-type mice (data not shown). We also found no evidence of hNGFR expression by dendritic cells, macrophages, neutrophils or a recently described, lineage-negative, Thy1^+^ population of innate lymphoid cells (Figure S3) [20]. We repeated these analyses in Smart-17A mice without the RORγt-GFP allele and also in mice colonized by segmented filamentous bacteria and obtained identical results (data not shown). In all cases, none of the examined cell types, including cells from intestinal tissues, constitutively expressed hNGFR, suggesting that IL-17A is not expressed or is expressed at levels too low to be detected using this reporter in resting mice.

### IL-17A expression during *Klebsiella pneumoniae* infection

*Klebsiella pneumoniae* is a gram-negative extracellular bacterium that is a cause of nosocomial and community-acquired pneumonia. IL-17A is rapidly produced in the lungs of mice during *K. pneumoniae* infection [21], and downstream signaling through the IL-17 receptor (IL-17R) leads to the induction of a variety of proinflammatory cytokines and chemokines that promote neutrophil accumulation [22]. Mice deficient in IL-17A, IL-17R or the p19 subunit of IL-23 have increased bacterial dissemination and increased mortality [22], [23], [24], supporting the use of this model to investigate the cell types that produce IL-17A during acute bacterial challenge.

**Figure 3:**
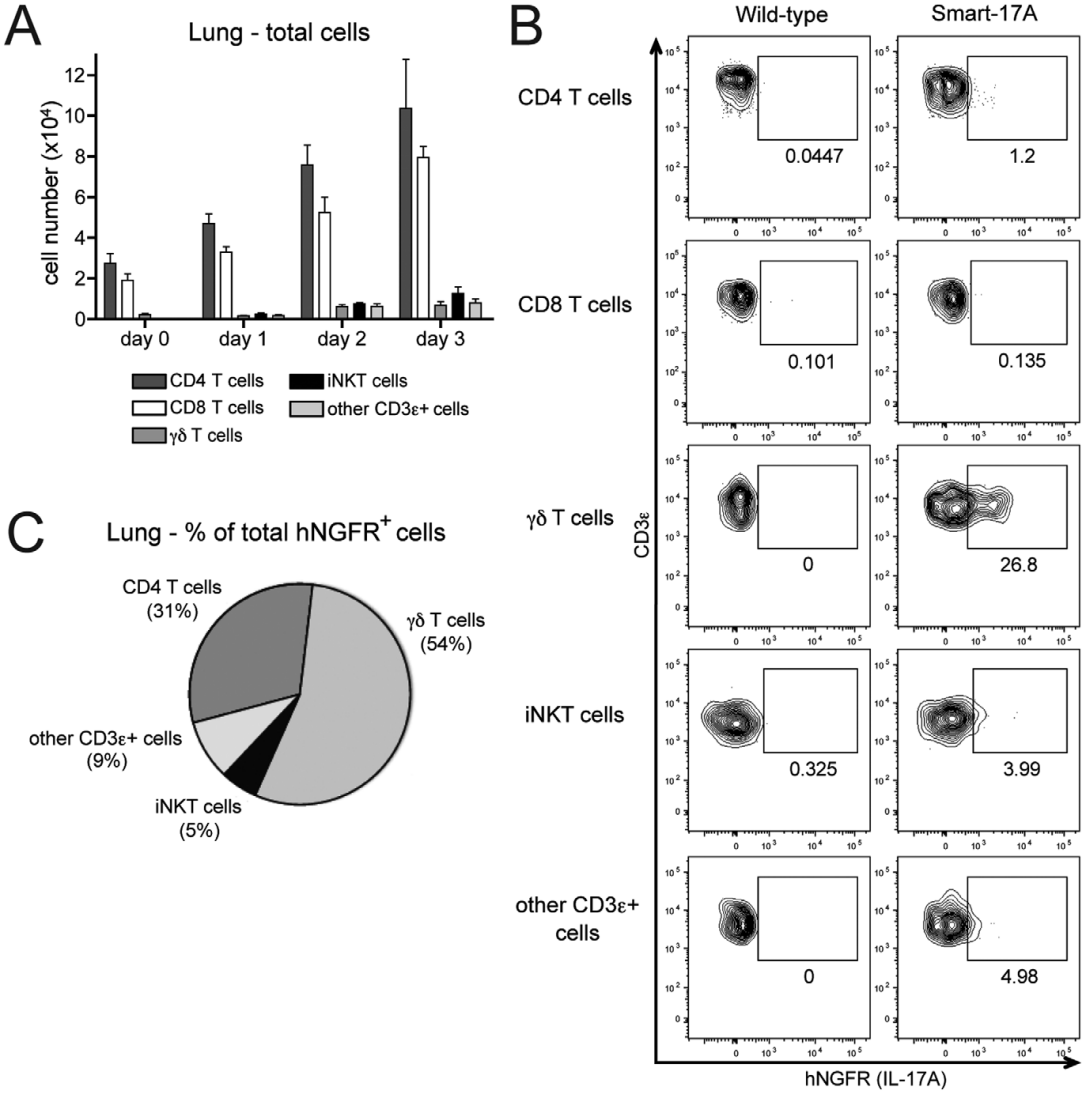
IL-17A expression during infection with *Klebsiella pneumoniae.* Wild-type or Smart-17A mice were infected with 500–1000 *K. pneumoniae*. (A) At the indicated time points, cells were harvested from lungs and numbers of cells were enumerated. (B) Cells were isolated from the lungs of mice 2 days after infection and assayed for hNGFR expression. (C) The total number of hNGFR^+^ and YFP^+^ cells on day 2 post-infection were calculated and the percentage attributable to each cell population is shown in a pie graph. The percentage of background staining seen in a wild-type mouse under identical conditions was subtracted before performing all calculations to control for nonspecific staining. This experiment was repeated 3 times with n >3 mice at each time point. For bar and pie graphs, data from independent experiments were compiled. For flow cytometry, representative plots are shown.

We intranasally inoculated Smart-17A mice with a dose of *K. pneumoniae* that led to mortality in a majority of infected mice by day 5. During the first 3 days after infection, we noted a substantial increase in the total number of CD3ε^+^ cells in the lungs (Figure 3A). The vast majority of these cells were CD4^+^ and CD8^+^ T cells, with much smaller numbers of γδ T cells, iNKT cells and other CD3ε^+^ cells. When assayed two days post-infection, the highest percentages of hNGFR^+^ cells were found amongγδT cells, with lower percentages of hNGFR^+^ iNKT cells and other CD3ε^+^ cells (Figure 3B). A small but reproducible percentage of CD4^+^ T cells were also hNGFR^+^, whereas percentages of reporter-positive CD8^+^ T cells did not differ from background levels in wild-type mice. We did not observe hNGFR expression by any CD3ε^-^ cell populations (data not shown). Although innate-like T cells comprised only a small portion of the total number of CD3ε^+^ cells in the infected lung, they represented a majority of the total hNGFR^+^ cells (Figure 3C). γδ T cells themselves comprised over half of the hNGFR^+^ cells (54%), followed by CD4^+^ T cells (31%), other CD3ε^+^ cells (9%) and iNKT cells (5%). Thus, innate-like T cells are major sources of IL-17A during *K. pneumoniae* infection.

### Innate-like T cells are poised to produce IL-17A in response to proinflammatory cytokines

**Figure 4:**
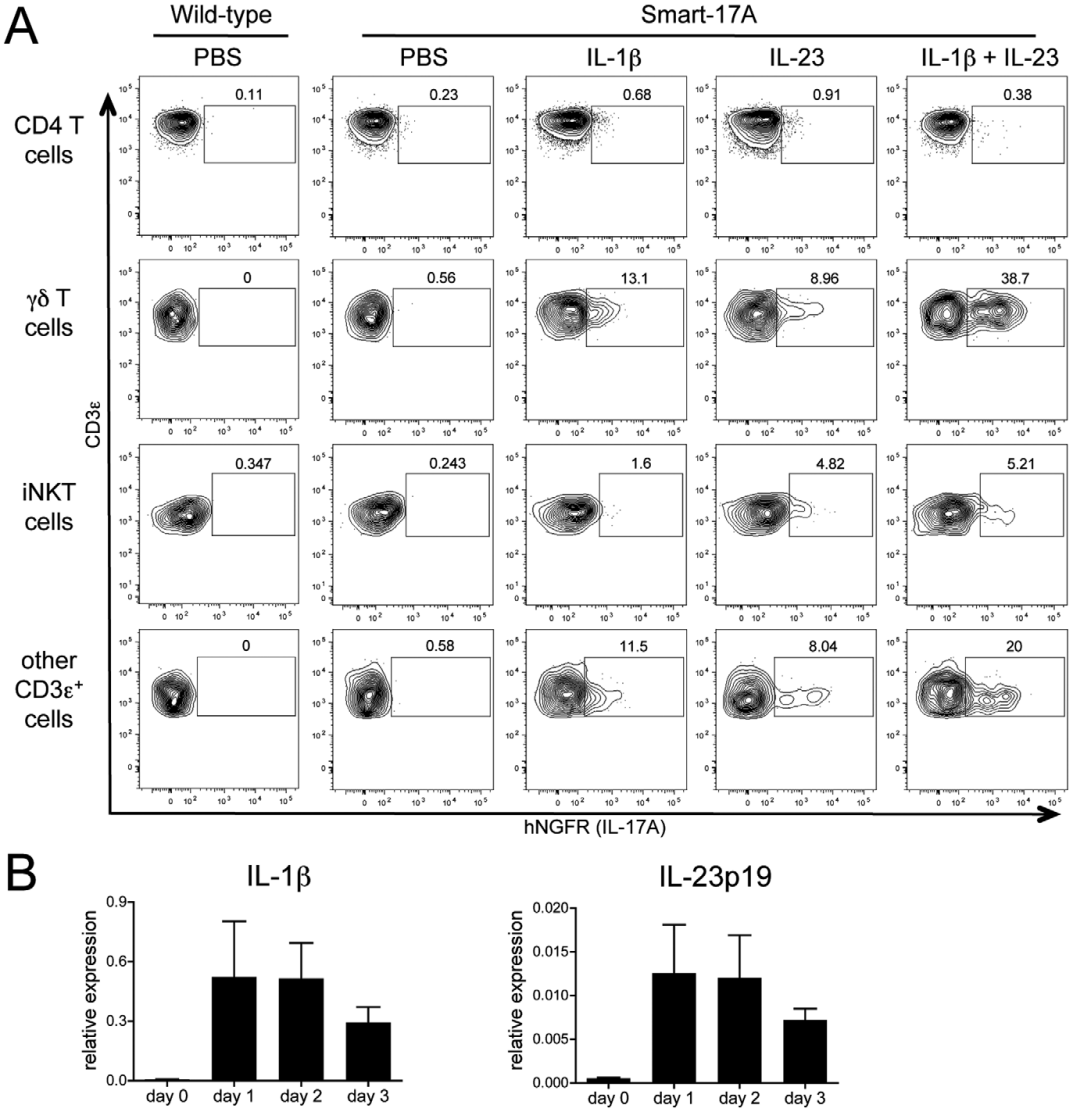
Expression of IL-17A from innate-like T lymphocytes cells can be induced by IL-1β and/or IL-23. (A) Smart-17A mice were inoculated intranasally with PBS or with 500 ng IL-1β, IL-23 or both cytokines. Lungs were harvested 8 hr later and cells were analyzed for hNGFR expression. Gates were set using a wild-type control. The experiment was repeated 2 times and representative data are shown. (B) Wild-type mice were infected with *K. pneumoniae* and levels of IL-1β and the IL-23 subunit p19 mRNA in whole lung homogenate were measured using quantitative PCR. Expression of GAPDH was used as a reference to define relative expression. The experiment was done twice and a representative experiment is shown, n = 3 for all groups.

The discovery that innate-like T cells expressed IL-17A during acute *K. pneumoniae* infection led us to investigate the potential signals that mediate the production of this cytokine. It has been noted that IL-1β and IL-23 can induce TCR-independent IL-17A production from γδ T cells *in vitro* [7] and TCR/CD1d-dependent IL-17A production from iNKT cells *in vitro* and *ex vivo* [8]. To determine if these findings applied to innate-like T lymphocytes *in vivo*, we administered IL-1β and IL-23 intranasally to Smart-17A mice and examined reporter expression from cells in the lungs 8 hr later (Figure 4A). The addition of either IL-1β or IL-23 individually elicited hNGFR expression in γδ T cells, iNKT cells and other CD3ε^+^ cells, and the combination of both cytokines resulted in a synergistic increase in hNGFR levels to levels similar to those seen after *K. pneumoniae* infection. In contrast, we did not observe induction of IL-17A production as assessed by hNGFR expression in CD4^+^ T cells. The brief time between cytokine administration and IL-17A expression suggests that tissue resident innate-like T cells are poised to produce IL-17A rapidly after receiving signals from these cytokines. IL-1β and IL-23 subunit p19 mRNA were induced in the lung within 1 day after *K. pneumoniae* infection (Figure 4B), providing further evidence that these cytokines may play a role in inducing acute IL-17A production from innate-like T lymphocytes.

### IL-17A expression during experimental autoimmune encephalomyelitis

Experimental autoimmune encephalomyelitis (EAE) is a mouse model of multiple sclerosis that is associated with the production of IL-17A. IL-17A^−/−^ mice have reduced incidence and severity of disease [25]. IL-1R^−/−^ mice and IL-23p19^−/−^ mice are also less susceptible to disease [7], [26]. Although Th17 cells are thought to be the main source of IL-17A during EAE, γδ T cells can also produce this cytokine and may act to amplify IL-17A production from Th17 cells [7], [14]. Most of these studies relied on *ex vivo* restimulation to assay IL-17A production, however, and thus the cells producing IL-17A directly *in vivo* during the development and propagation of EAE are not fully characterized.

**Figure 5:**
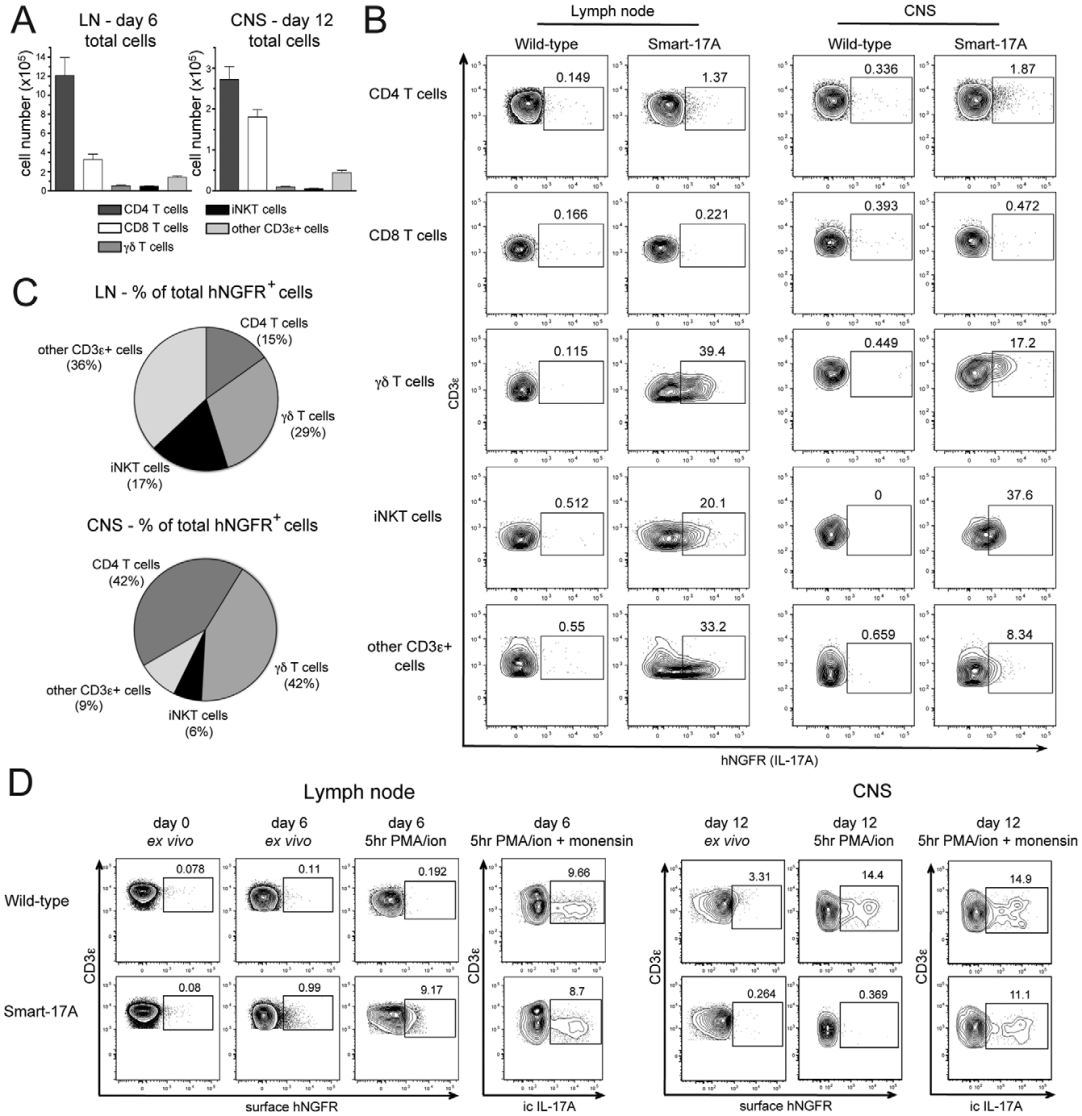
IL-17A expression during experimental autoimmune encephalomyelitis. Wild-type or Smart-17A mice were immunized with MOG-CFA to induce EAE. (A) At the indicated time point, cells were harvested from the draining axial, brachial and inguinal lymph nodes (LN) at day 6 or spinal cords and cerebellums (CNS) at day 12 and numbers of cells were enumerated. (B) Cells were assayed for hNGFR expression. (C) The total numbers of hNGFR^+^ cells were calculated and the percentage attributable to each cell population is shown in a pie graph. The percentage of background staining seen in a wild-type mouse under identical conditions was subtracted before performing all calculations to control for nonspecific staining. (D) Cells were isolated from the LN or CNS and immediately stained for surface markers or restimulated for 5 hr with PMA/ionomycin and then stained for surface markers and/or intracellular expression of IL-17A. The experiments in A–C were repeated 3 times with n>2 mice at each time point. The experiment in D was repeated 2 times with n>5 mice at each time point. For bar and pie graphs, data from independent experiments were compiled. For flow cytometry, representative plots are shown. For the CNS data, mice were excluded that did not display symptoms of paralysis.

To track IL-17A expression during EAE, we immunized Smart-17A mice with myelin oligodendrocyte glycoprotein peptide (MOG) emulsified in complete Freund's adjuvant (CFA) and treated mice with pertussis toxin to induce disease. We then examined CD3ε^+^ cell lineages in the draining lymph nodes (LN) at day 6 and in the spinal cord and cerebellum (referred to as CNS) at day 12 when mice displayed symptoms of paralysis. At both sites, the vast majority of isolated T cells were CD4^+^ or CD8^+^ T cells, with only small numbers of γδ T cells, iNKT cells and other CD3ε^+^ cells (Figure 5A). Small but reproducible percentages (1–2%) of CD4^+^ T cells expressed the hNGFR reporter in both the LN and the CNS (Figure 5B). Among the remaining T cell populations, we observed hNGFR-expressing γδ T cells, iNKT cells and other CD3ε^+^ cells (Figure 5B). The percentages of these hNGFR^+^ innate-like T cells were greater in the LN than the CNS. Expression of hNGFR by CD8^+^ T cells in both the LN and CNS was negligible when compared to wild-type controls, and we did not observe hNGFR expression by any CD3ε^−^ cells (data not shown). Although the total numbers of γδ T cells, iNKT cells and other CD3ε^+^ cells were substantially lower than the total numbers of CD4^+^ cells, these innate-like T cells comprised the majority of IL-17A-expressing hNGFR^+^ cells in both the LN and CNS (Figure 5C). In the LN, the greatest fraction of hNGFR^+^ cells was other CD3ε^+^ cells (36%), followed by γδ T cells (29%), iNKT cells (17%) and CD4^+^ T cells (15%). In the CNS, the largest fractions of hNGFR^+^ cells were γδ T cells and CD4^+^ T cells (42% each), while other CD3ε^+^ cells and iNKT cells together made up the remaining 16%. These data suggest that innate-like T cells are a significant source of IL-17A during both the initiation and propagation phases of EAE.

Since the percentages of IL-17A-expressing CD4^+^ T cells observed using the Smart-17A reporter were substantially lower than what has been reported in the literature using *ex vivo* restimulation, we verified that *ex vivo* restimulation of cells isolated from the LN and CNS of Smart-17A mice led to an induction of hNGFR expression that very closely mirrored the percentages of IL-17A^+^ cells found using standard intracellular cytokine staining methods (Figure 5D). This confirms that cells from Smart-17A mice accurately report IL-17A production, and further suggests that *ex vivo* restimulation can induce IL-17A production from cells that are not actively producing the cytokine *in vivo*.

### Differential production of IL-17A and IFN-γ at effector sites during inflammation

IFN-γ is a cytokine that is expressed by multiple cell types and is generally associated with inflammatory immune responses. Mice deficient in IFN-γ or the IFN-γ receptor (IFN-γR) are more susceptible to a variety of bacterial infections including pulmonary infection with *K. pneumoniae* [27]. IFN-γ has also been implicated in the pathogenesis of autoimmune diseases, including EAE, although the precise role of this cytokine is controversial. Thus, mice deficient in IFN-γ, IL-12 p35 subunit and IFN-γR are more susceptible to disease [28], [29], [30] whereas T-bet^−/−^ mice are protected from disease [31]. EAE can be induced in Rag^−/−^ mice by the transfer of CD4^+^ T cells that have been polarized under either Th1 or Th17 conditions [32]. Complicating matters further, recent studies have suggested that Th17 cells may represent an unstable population that can convert to an IFN-γ producing phenotype under specific inflammatory conditions [33].

**Figure 6:**
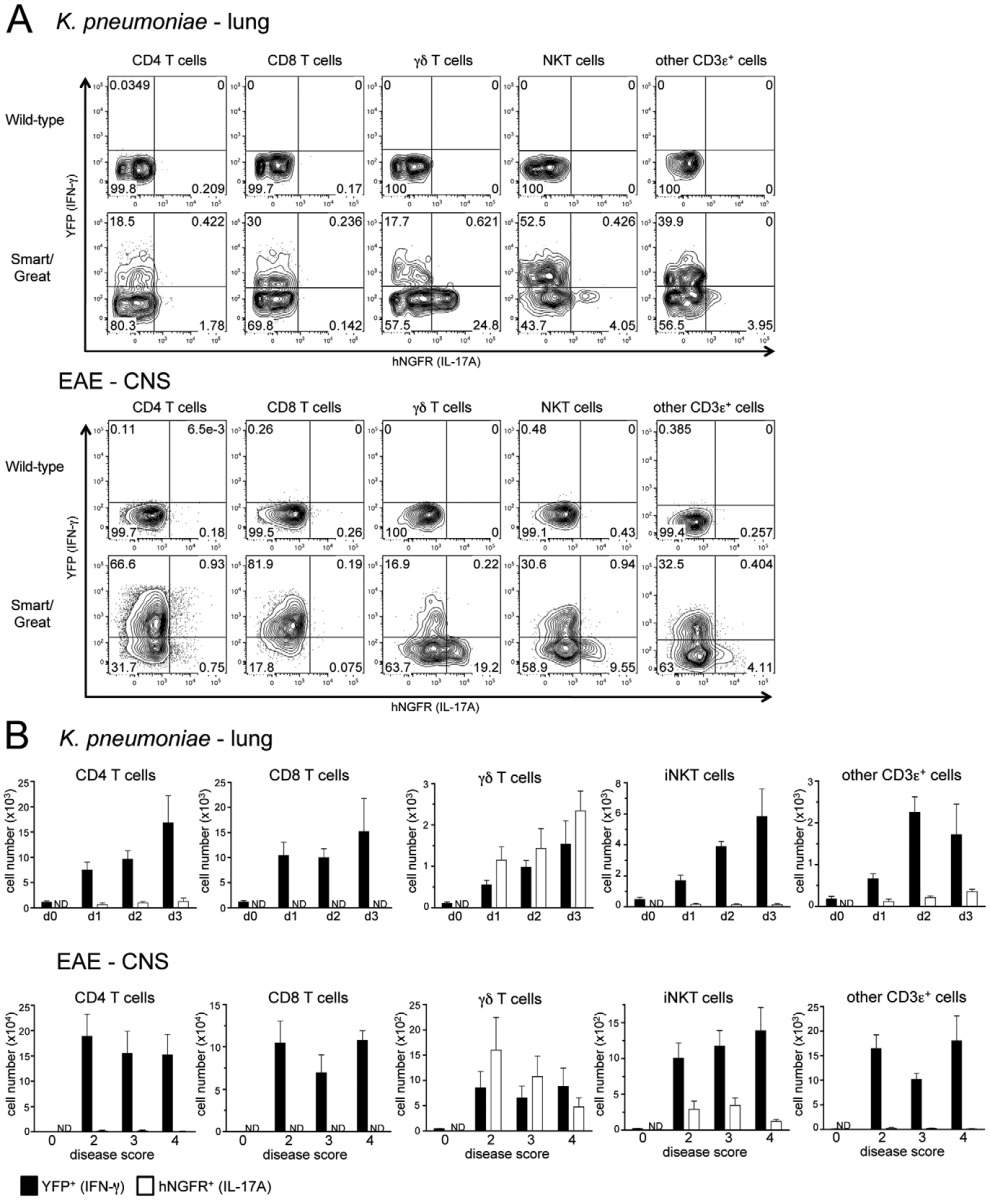
Differential production of IL-17A and IFN-γ in effector sites during inflammation. (A) Expression of hNGFR and YFP was assessed in the lungs of mice at day 2 after infection with *K. pneumoniae* or in the spinal cord and cerebellum (CNS) of mice at day 12 after induction of EAE. (B) Numbers of hNGFR^+^ and YFP^+^ cells were enumerated during *K. pneumoniae* infection or EAE disease course. EAE disease scores were determined as described in Materials and Methods. ND  =  not detected. These experiment was repeated 3 times with n>2 mice at each time point or disease score. For flow cytometry, representative plots are shown. For bar graphs, data from individual experiments were compiled.

To explore the relationship between IL-17A and IFN-γ production during *K. pneumoniae* infection and EAE, we crossed Smart-17A mice to Great reporter mice, which mark IFN-γ production by the coordinate expression of YFP downstream of an IRES introduced into the IFN-γ locus [34]. The most striking observation in these dual reporter mice was that at the effector sites following both challenges, either in the lungs of mice infected with *K. pneumoniae* or in the CNS of mice during EAE, the percentages of YFP^+^ cells were substantially greater than the percentages of hNGFR^+^ cells among CD4^+^ T cells, iNKT cells and other CD3ε^+^ cells (Figure 6A). In contrast, the populations of cells that expressed YFP and hNGFR was much more comparable among γδ T cells. As noted previously, we saw no measurable expression of hNGFR above background levels in CD8^+^ T cells, although we did note a substantial population of YFP^+^ CD8^+^ T cells, especially in the CNS during EAE. We observed some co-expression of YFP and hNGFR among CD4^+^ T cells, especially in the CNS of mice during EAE, where approximately half of the hNGFR^+^ cells also expressed YFP. However, among the innate-like T cells, IFN-γ-expressing and IL-17A-expressing cells segregated into distinct populations. The same trends in cytokine production were evident when we examined the total numbers of YFP^+^ and hNGFR^+^ cells isolated from these effector sites (Figure 6B). IFN-γ-producing cells greatly outnumbered IL-17A-producing cells during the first three days of *K. pneumoniae* infection and during all stages of progressive EAE disease among all subsets of CD3ε^+^ cells except for γδ T cells.

## Discussion

We used IL-17A reporter mice to assess IL-17A expression in resting mice and during models of bacterial pneumonia and autoimmune disease. We did not observe constitutive IL-17A expression from cells in naïve mice. However, in appropriate infectious and autoimmune models, we observed IL-17A-expressing CD4^+^ T cells and greater percentages of IL-17A-expressing γδ T cells, iNKT cells and other CD3ε^+^ cells. The other CD3ε^+^ cells were defined by their expression of CD3ε^+^ and lack of expression of CD4, CD8, γδ TCR and the CD1d-tetramer. It is possible that these cells are type II NKT cells that do not recognize α-galactosylceramide, or T cells that have downregulated the expression of CD4, CD8 or the γδ TCR. In all of these studies, IL-17A production as assessed by the reporter was limited to CD3ε^+^ T cells and was not expressed by myeloid cell populations. By using novel dual cytokine reporter mice, we demonstrated that IFN-γ-expressing lymphocytes greatly outnumbered IL-17A-expressing lymphocytes in both the acute bacterial and in the more subacute autoimmune challenges.

We were unable to detect expression of the hNGFR reporter in any CD3ε^+^ or CD3ε^−^ cell type in naïve mice, suggesting that IL-17A is not expressed *in situ* in resting mice. Segmented filamentous bacteria (SFB), *Candidatus arthromitis*, have been shown to induce IL-17A production by lamina propria T cells when introduced into the mouse intestinal flora [35]. Cohousing Smart-17A mice with mice obtained from Taconic Farms led to colonization by SFB as determined by PCR, but did not induce hNGFR expression on cells in the lamina propria of the small intestine or colon (data not shown). Our inability to quantitate active IL-17A production from cells in tissues of resting mice contrasts with prior observations made with other strains of IL-17A reporter mice. In the first report, Hirota et al. [14] crossed IL-17A-cre mice to Rosa-flox-stop-YFP mice to generate an IL-17A fate-tracking reporter and observed that 11% of lamina propria CD4^+^ T cells were constitutively YFP-positive in these mice. A study by Esplugues et al. [15] using a second IL-17A reporter strain, which utilized an IRES-eGFP knockin strategy more closely akin to the construct used in the Smart-17A mice, reported that 2–4% of lamina propria CD4^+^ T cells constitutively expressed eGFP. Whether the differences between our results and those using these alternate reporter strains are technical (e.g. permissiveness of the various IRES elements or detection limits of eGFP versus hNGFR) is unclear, and will require direct comparisons of the strains in question.

Here, we crossed our Smart-17A reporter mice to RORγt-GFP reporter mice to ensure our capacity to isolate potential IL-17A-producing cells from the relevant tissues. Although we readily identified populations of RORγt^+^ cells in multiple organs of naïve mice, these cells did not constitutively express the hNGFR reporter. In line with a previous report [36], we noted substantial populations of RORγt^+^ γδ T cells in the skin and lungs of resting mice. We also noted smaller percentages of RORγt^+^ iNKT cells in the lung and other CD3ε^+^ cells in the lung and skin. Resident dermal RORγt^+^ γδ T cells have recently been shown to play a role in cutaneous immunosurveillance and to contribute to skin pathology during a mouse model of psoriasis [37], [38], [39]. The localization of constitutive RORγt^+^ innate-like T lymphocytes to the lung is intriguing as this mucosal tissue is relatively sterile and devoid of large numbers of commensal bacteria, a feature that is may contribute to the development of the RORγt^+^ populations in the intestinal tract [35], [40], [41]. Although lung-resident innate-like T cells did not constitutively express IL-17A as assessed by the hNGFR reporter, γδ T cells, iNKT cells and other CD3ε^+^ cells expressed hNGFR within 24 hr after *Klebsiella* infection or 8 hr after administration of IL-1β and/or IL-23. Taken together, these data suggest that RORγt^+^ innate-like T cells accumulate at multiple epithelial barriers, where they are poised to respond rapidly to compromises in epithelial integrity with IL-17A production and the subsequent recruitment of neutrophils to the injured site.

Earlier reports used *ex vivo* restimulation to identify Th17 cells induced during a variety of bacterial and fungal infections, particularly those initiated by mucosal challenges [42], [43], [44], [45], [46]. However, as we demonstrate here, *ex vivo* restimulation has the potential to reveal IL-17A production by cells that may not be actively producing this cytokine *in vivo* (Figure 5D). Although our studies using *K. pneumoniae* in Smart-17A mice revealed activation of reporter expression in both innate-like T cells and CD4^+^ Th17 cells, earlier studies demonstrated increased mortality in mice lacking γδ T cells but no difference in mortality in mice lacking αβT cells [22], [46], suggesting that γδ T cells may be a more important source of IL-17A during this infection. Similar observations were made in experimental *Mycobacterium tuberculosis* infection [47] and in intraperitoneal infection with *Escherichia coli* [48], suggesting a more generalized role for IL-17A production by γδ T cells in immunity to bacterial infections. Our findings corroborate these interpretations.

Somewhat unexpected were the small percentages of IL-17A-expressing CD4^+^ T cells recovered from lymph nodes and spinal cords and cerebellums during the peak of EAE disease, a canonical experimental model for Th17 induction. Despite the relatively small percentage of IL-17A reporter-positive CD4^+^ T cells, the large numbers of CD4^+^ T cells in the inflammatory milieu was such that CD4^+^ T cells still comprised a sizeable portion of total IL-17A-producing cells, making up 15% of the total in the LN and 42% in the CNS. The vast majority of effector CD4^+^ T cells, however, were Th1 cells as assessed by expression of the Great IFN-γ reporter allele and, based on our *in vitro* studies of the decay of the reporter on Th17 cells, are unlikely to have secreted IL-17A over the prior 1–2 days. Our findings seem to contrast with those obtained using an IL-17A-cre/ ROSA-flox-stop-YFP fate-tracking reporter mouse (14). In that study, the authors observed that over half of the CD4^+^ and γδ T cells were YFP^+^ in the spinal cords of mice during the peak of EAE. However, cells positive for the IL-17A-cre/Rosa-flox-stop-YFP reporter were those that expressed the Cre recombinase from an activated IL-17A locus at any time during their development, whereas the Smart-17A reporter specifically marks cells that have recently expressed or are currently expressing IL-17A. Thus, differences in construction of these various reporter mice may account for the differences in the experimental results.

Recent reports have raised the possibility that Th17 cells represent an unstable transient phenotype rather than a fully differentiated Th subset akin to that of Th1 and Th2 cells. During the development of EAE, IL-17A^+^, IFN-γ^+^ and IL-17A/IFN-γ double-positive CD4^+^ T cells have been observed in the lymph nodes and spinal cords of immunized mice [19]. Adoptive transfer of encephalitogenic Th17 cells purified from an IL-17F-cre BAC transgenic mouse into RAG-2^−/−^ or wild-type recipients revealed that a portion of these cells began to secrete IFN-γ during the progression of EAE [49]. Similarly, studies using the IL-17A-cre/Rosa-flox-stop-YFP mice revealed that only half of the reporter-positive fate-marked CD4^+^ T cells were positive for intracellular IL-17A in the spinal cords during the peak of EAE [14], providing further evidence for a switch from an IL-17A-producing to an IFN-γ-producing phenotype. However, all of these studies used *ex vivo* restimulation to assess IL-17A and IFN-γ production from isolated spinal cord cells. Our study, which relied solely on direct *ex vivo* detection of cytokine production using knockin reporter mice, provides additional evidence for the marked predominance of IFN-γ production by CD4^+^ T cells during each of the clinical stages of EAE. We saw only low numbers of IL-17A^+^ cells as compared to IFN-γ^+^ cells, and approximately half of the Th17 cells concordantly expressed IFN-γ. Taken together, these findings suggest that activation of the IL-17A locus may be important in the early differentiation of pathogenic CD4^+^ T cells in EAE, but that actual production of IL-17A might not be a major mechanism driving the neurological manifestations of the disease. Indeed, recent reports have demonstrated critical contributions by GM-CSF rather than IL-17 in the pathogenesis of EAE [50], [51].

A consistent observation was the pronounced segregation of IL-17A-expressing and IFN-γ-expressing cells within the innate-like T cell populations. This segregation occurred in both the infectious and autoimmune models, raising questions about the mechanisms of regulation that account for this exclusionary pattern of cytokine production. IFN-γ and IL-17 have been shown to limit differentiation of Th17 and Th1 cells, respectively, and this mutual inhibition could be operating in these innate populations as well [52], [53], [54]. It has also been suggested that antigen-naive γδ T cells predominately produce IL-17A when activated, whereas antigen-experienced γδ T cells produce IFN-γ [55], which could potentially explain the differences in cytokine-secreting populations seen in our models. Further characterization of the cells expressing these cytokines is needed to address this question more fully.

Cytokine reporter mice allow for the functional marking of cells during the course of an inflammatory challenge and have provided essential insights into the coordination of the immune response *in vivo*. The Smart-17A mice and Great mice used in this study permitted the detection of IL-17A-producing and IFN-γ-producing cells *in situ* without restimulation. Using these mice, we demonstrated that innate-like T cells, particularly γδ T cells, comprised major cell populations poised for acute IL-17A production. During both *K. pneumoniae* infection and EAE, models previously demonstrated to induce potent IL-17 expression, we show that the numbers of IL-17A-producing cells were far fewer than the numbers of IFN-γ-producing cells in the same tissues, suggesting different levels of regulation of these two inflammatory cytokines *in vivo*. Although IL-17A production has been elicited from a number of different cell types using restimulation, our reporter system suggests that cytokine production is limited to T cells in the models we studied. These cytokine reporter mice will be valuable tools for future studies investigating the full contributions of IL-17A-expressing cells to vertebrate immunity.

## Materials and Methods

### Mice

C57BL/6 mice and RORγt-GFP reporter mice [18] were obtained from Jackson Laboratories. Great IFN-γ reporter mice have been described [34]. Smart-17A mice were generated by first assembling a composite selection/reporter cassette using standard cloning procedures in the following order: 1) a floxed-neomycin-resistance gene (floxed Neo^r^); 2) encephalomyocarditis virus (EMCV) internal ribosome entry site (IRES); 3) low-affinity human nerve growth factor receptor (p75 LNGFR, also known as CD271) cDNA (OriGene); 4) bovine growth hormone (bGH) poly-adenylation signal (pA). This 3.1 kb selection/reporter cassette was cloned into a basal targeting construct pKO915-DT (Lexicon) containing diphtheria toxin (DT)α chain for negative selection. Both 5′ and 3′ homologous arms used to flank the cassette were obtained by high-fidelity PCR amplification of the *il17a* locus from 129/SvJ genomic DNA. The 5′ arm consists of a 1.6 kb fragment covering intron 2 and coding sequence of exon 3. The 3′ arm consists of a 2.2 kb fragment spanning the endogenous 3′ UTR and downstream sequences. After sequence verification, the NotI-linearized construct was electroporated into PrmCre ES cells, which express Cre recombinase driven by the protamine promoter [56]. G418-resistant ES clones were screened for homologous recombination by Southern blot. Two independent clones were injected into C57BL/6 blastocysts to generate chimeras. The neomycin-resistance cassette was deleted in the male germline by Cre-mediated recombination after breeding male chimeras to C57BL/6 females. Mice carrying the Smart17A allele were backcrossed 10 generations to the C57BL/6 background. In experiments utilizing Smart-17A/RORγt-GFP mice, the mice were homozygous for the Smart-17A allele and heterozygous for RORγt-GFP. Smart-17A/Great mice were homozygous for both the IL-17A and IFN-γ reporter alleles and these dual reporter mice were used for all experiments described in Figures 3, 4, 5, and 6.

### Th17 polarization

CD4^+^ T cells were isolated from the lymph nodes of Smart-17A and wild-type C57BL/6 mice using MACS beads (Miltenyi Biotech) and cultured with irradiated splenocytes from TCR-Cα*^−/−^* mice. Cells were stimulated under the designated conditions for 4 days: Th0 (50 U/ml IL-2), Th1 (50 U/ml IL-2, 5 ng/ml IL-12, 10 µg/ml anti-IL-4), Th2 (50 U/ml IL-2, 50 ng/ml IL-4, 10 µg/ml anti-IFN-γ), Th17 (3 ng/ml TGF-β, 20 ng/ml IL-6, 10 µg/ml anti-IFN-γ and anti-IL-4). Cytokines were purchased from R&D Systems. For intracellular cytokine staining, cells were restimulated with phorbol myristate acetate (50 ng/ml) and ionomycin (750 ng/ml) for 5–6 hr, with monensin (3 µM) added for the final 2 hr.

### *Klebsiella pneumoniae* infection

*K. pneumoniae* (American Type Culture Collection #43816) were cultured in Nutrient Broth (Difco) with shaking overnight at 37°C. Cultures were diluted in Nutrient Broth and cultured for an additional 2–3 hr until bacteria reached log phase. Bacteria were pelleted by centrifugation, washed twice in PBS and diluted to a final dose of 500–1,000 bacteria in 50 µl PBS. This inoculum was administered intranasally after anaesthetizing mice with isofluorane. Doses were confirmed by plating the inoculum on Nutrient Broth agar plates and counting colonies the following day.

### Induction of experimental autoimmune encephalomyelitis

Mice were immunized with 200 μg of MOG35–55 emulsified in CFA containing 4 mg/ml *Mycobacterium tuberculosis* (Difco) subcutaneously and given 200 ng of pertussis toxin (List Biological Laboratories) intravenously on the day of and 2 days after immunization. Animals were graded daily according to their clinical severity as follows: grade 0, no abnormality; grade 1, limp tail; grade 2, limp tail and hind limb weakness (waddling gait); grade 3, partial hind limb paralysis; grade 4, complete hind limb paralysis; grade 5, moribund.

### Cell isolation and preparation

Mice were perfused with PBS and organs were removed. Mechanical dissociation was used to prepare single-cell suspensions from lymph nodes, spleens and Peyer's patches. Lungs were dissociated using a GentleMacs Dissociator (Miltenyi Biotech). Small intestines and colons were cut into small pieces and incubated in 5 mM EDTA in HBSS on stir plates 4 times for 15 min to remove the epithelial layer containing intraepithelial lymphocytes. Intestinal pieces were incubated at 37°C in 200 U/ml collagenase VIII (Sigma) in complete RPMI for a total of 4 30-min incubations. Cells from all incubations were pooled and lamina propria lymphocytes were purified over a 40%/100% Percoll gradient. Central nervous system lymphocytes were isolated as described (21) with modifications. Briefly, spinal cords and cerebellums were cut into pieces and digested in 300 U/ml Mandl units Collagenase D (Roche) and 50 U/ml DNase I (Roche) at 37°C for 30 min. Lymphocytes were enriched by separation on a 30%/70% Percoll gradient.

### Flow cytometry

Single-cell suspensions were washed in FACS buffer (PBS, 3% FCS, 1 mg/L NaN_3_), and the cell pellets were incubated for 10 min on ice with anti-CD16/CD32 monoclonal antibodies (UCSF Antibody Core Facility). Cells were incubated for 30 min on ice with antibodies to surface markers. As necessary, cells were washed and stained with secondary antibodies for an additional 20 min on ice. Live cells were gated using DAPI exclusion. For intracellular cytokine staining, cells were stained for surface markers, fixed in 2% formaldehyde in PBS for 20 min at room temperature, washed and permeabilized in FACS buffer plus 0.5% saponin. Cells were stained at room temperature for 30 min in buffer containing 0.5% saponin buffer and 25% FCS. Dead cells were excluded using a violet live/dead fixable stain (Invitrogen). Antibodies were as designated and included: CD4 (BD, eBioscience), CD8 (BD, Biolegend), γδ (eBioscience), CD3ε (eBioscience), CD11b (BD, Biolegend), CD19 (Biolegend), Gr1 (BD), CD11c (BD), Thy1.2 (eBioscience), Sca-1 (BD), IL-17A (BD, eBioscience), hNGFR (LabVision), Streptavidin-PE (Invitrogen), Streptavidin-APC (Biolegend). PBS-157-loaded CD1d tetramer was obtained from the NIH Tetramer Core Facility. Cell counts were performed using Count-Bright absolute counting beads (Invitrogen). Samples were acquired on a LSRII flow cytometer (BD Biosciences) and analyzed using FlowJo software (Tree Star).

### Quantitative PCR

Whole lungs were homogenized and RNA was isolated using RNAzol (Molecular Research Center, Inc.). cDNA was prepared using the SuperScript III First Strand Synthesis System (Invitrogen). Primer sequences (PrimerBank) where designated were as follows: IL-1β: GAAATGCCACCTTTTGACAGTG, CTGGATGCTCTCATCAGGACA; IL-23p19: CAGCAGCTCTCTCGGAATCTC, GCATGTGCGTTCCAGGCTA. Transcripts were quantified by incorporation of SYBR Green (Invitrogen) on a StepOne Plus Real-Time PCR System (Applied Biosystems) and quantified relative to the expression of *GAPDH* (glyceraldehyde 3-phosphate dehydrogenase).

## Supporting Information

Figure S1 **Polarization of Smart-17A CD4 T cells** ***in vitro.*** (A) CD4^+^ T cells were isolated from Smart-17A mice using MACS beads and polarized under Th0, Th1, Th2 or Th17 conditions for 4 days, at which point surface hNGFR expression was assayed by flow cytometry. This experiment was repeated 3 times and representative flow cytometry plots are shown. (B) CD4^+^ T cells from wild-type or Smart-17A mice were polarized under Th17 conditions for 4 days. Cells were restimulated with PMA and ionomycin and then washed and re-plated in wells containing no cytokines. The percentage of hNGFR^+^ cells were measured at indicated time points to determine the rate of decay of the hNGFR reporter. A representative graph is shown from two comparable experiments.
(TIF)

Figure S2 **Gating of CD3ε^+^ cell populations.** Flow cytometry gating schemes for CD3ε^+^ cells used throughout this study. (A) Gating scheme for CD4^+^ T cells, γδ T cells, iNKT cells and other CD3ε^+^ cells. (B) Gating scheme for CD8^+^ T cells. Plots shown are from the mesenteric lymph node of a naïve Smart-17A mouse.
(TIF)

Figure S3 **IL-17A expression in CD3ε^−^ cell populations.** Cells were isolated from the indicated organs of Smart-17A/RORγt dual reporter mice and assayed for GFP and surface hNGFR expression. Dendritic cells were defined as CD11c^+^, macrophages as CD11b^+^, neutrophils as CD11b^+^ and Gr1^+^, and innate lymphoid cells as lineage-negative (negative for CD3ε, CD8, CD19, CD11b, Gr1) and Thy1^+^. The gated innate lymphoid cells included cells that were positive and negative for both CD4 and Sca-1. hNGFR expression was not seen using any gating scheme. All gates were drawn using a wild-type mouse as a control. The experiment was repeated twice and representative plots are shown.
(TIF)
