# Supplementary figures and images for: Marking and Quantifying IL-17A-Producing Cells In Vivo
Source: PLoS One. 2012 Jun 29;7(6):e39750. doi: 10.1371/journal.pone.0039750 (PMC3387253; doi:10.1371/journal.pone.0039750)

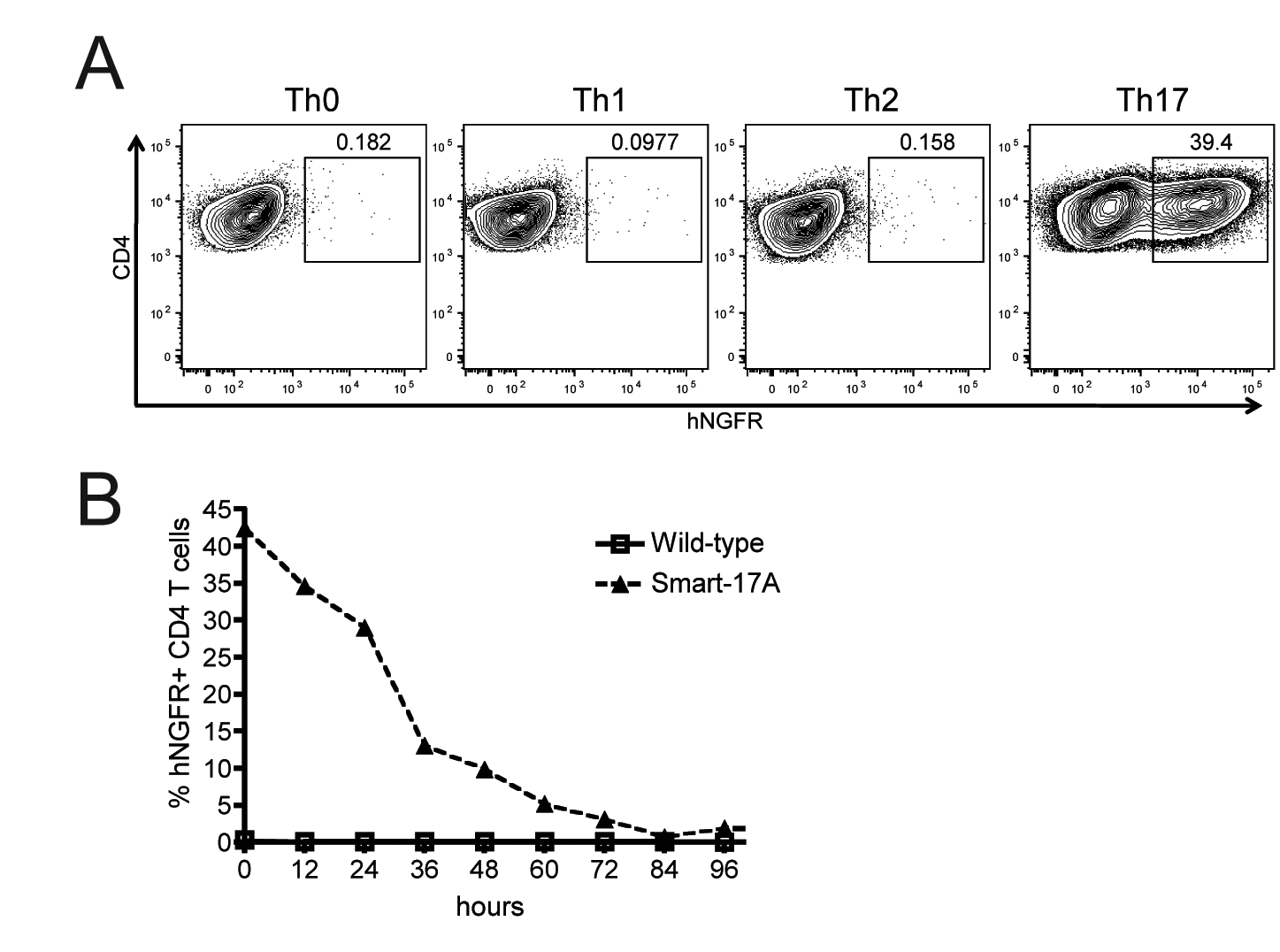

Supplement: Figure S1 — Polarization of Smart-17A CD4 T cells in vitro. (A) CD4+ T cells were isolated from Smart-17A mice using MACS beads and polarized under Th0, Th1, Th2 or Th17 conditions for 4 days, at which point surface hNGFR expression was assayed by flow cytometry. This experiment was repeated 3 times and representative flow cytometry plots are shown. (B) CD4+ T cells from wild-type or Smart-17A mice were polarized under Th17 conditions for 4 days. Cells were restimulated with PMA and ionomycin and then washed and re-plated in wells containing no cytokines. The percentage of hNGFR+ cells were measured at indicated time points to determine the rate of decay of the hNGFR reporter. A representative graph is shown from two comparable experiments. (TIF) [file pone.0039750.s001.tif]

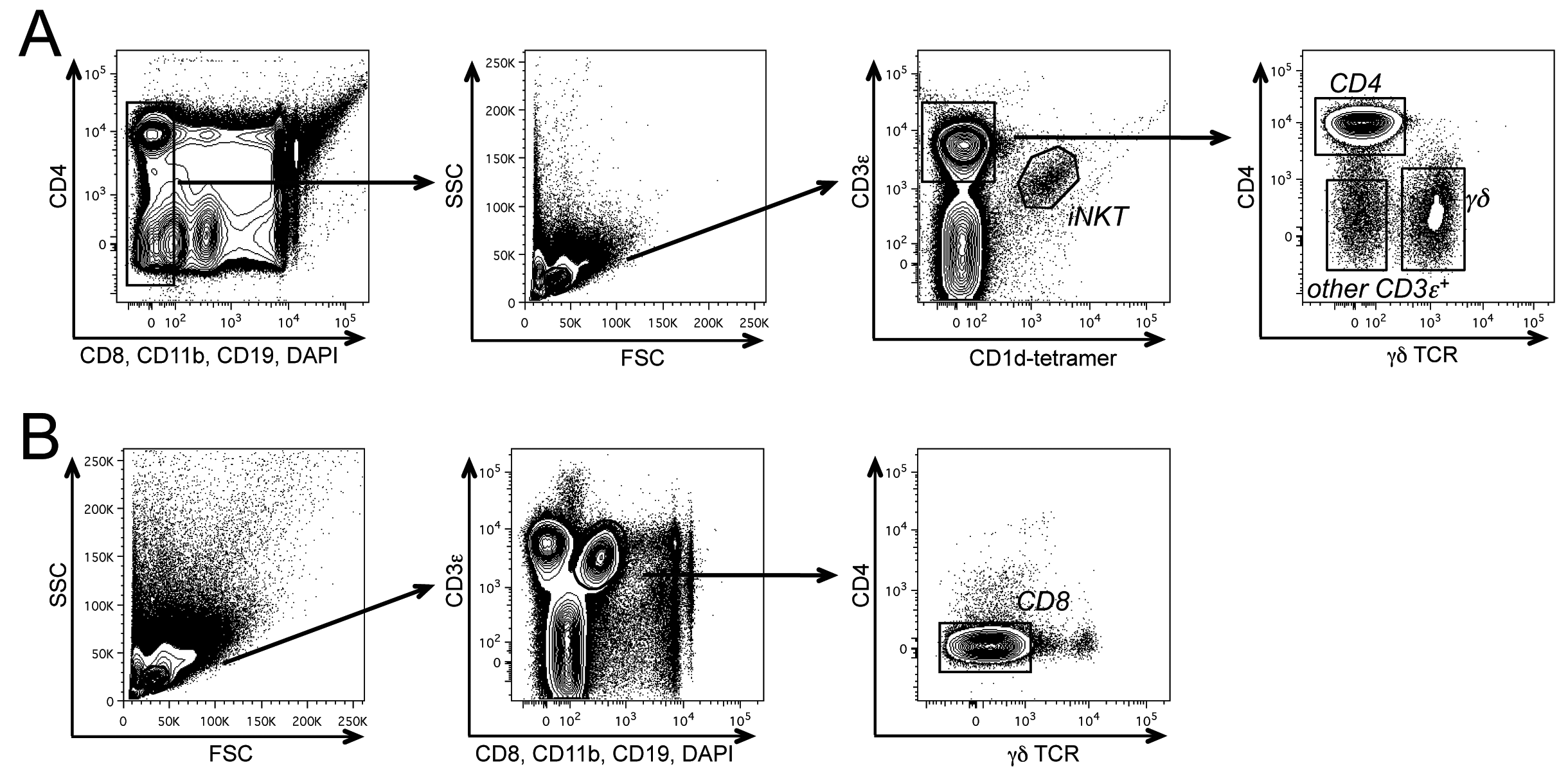

Supplement: Figure S2 — Gating of CD3ε+ cell populations. Flow cytometry gating schemes for CD3ε+ cells used throughout this study. (A) Gating scheme for CD4+ T cells, γδ T cells, iNKT cells and other CD3ε+ cells. (B) Gating scheme for CD8+ T cells. Plots shown are from the mesenteric lymph node of a naïve Smart-17A mouse. (TIF) [file pone.0039750.s002.tif]

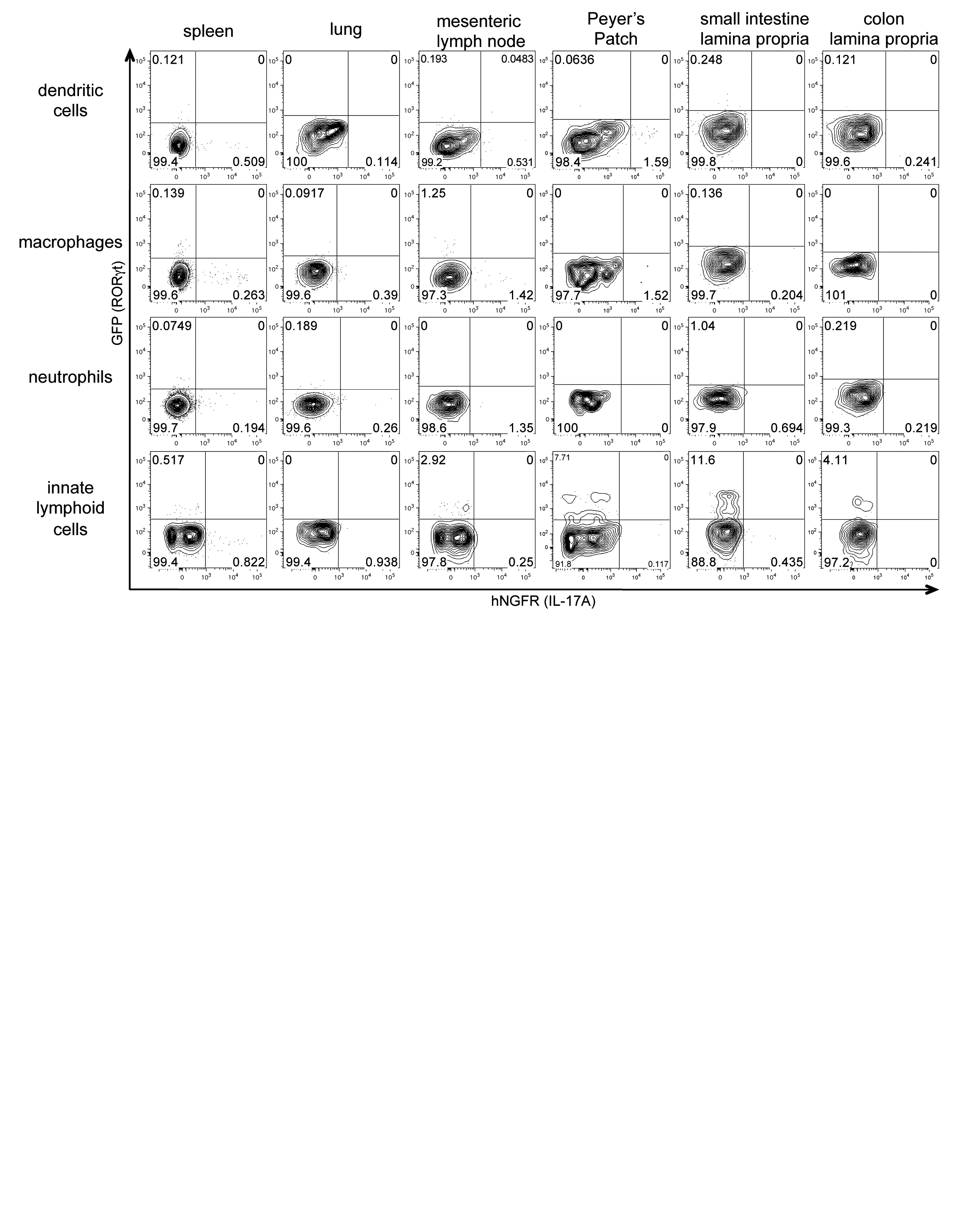

Supplement: Figure S3 — IL-17A expression in CD3ε− cell populations. Cells were isolated from the indicated organs of Smart-17A/RORγt dual reporter mice and assayed for GFP and surface hNGFR expression. Dendritic cells were defined as CD11c+, macrophages as CD11b+, neutrophils as CD11b+ and Gr1+, and innate lymphoid cells as lineage-negative (negative for CD3ε, CD8, CD19, CD11b, Gr1) and Thy1+. The gated innate lymphoid cells included cells that were positive and negative for both CD4 and Sca-1. hNGFR expression was not seen using any gating scheme. All gates were drawn using a wild-type mouse as a control. The experiment was repeated twice and representative plots are shown. (TIF) [file pone.0039750.s003.tif]
